# Lactate oxidation facilitates growth of *Mycobacterium tuberculosis* in human macrophages

**DOI:** 10.1038/s41598-017-05916-7

**Published:** 2017-07-25

**Authors:** Sandra Billig, Marie Schneefeld, Claudia Huber, Guntram A. Grassl, Wolfgang Eisenreich, Franz-Christoph Bange

**Affiliations:** 1 Dept. of Medical Microbiology and Hospital Epidemiology, Hannover Medical School, Hannover, 30625 Germany; 2 Dept. of Biochemistry, Technische Universität München, Garching, 85747 Germany; 3 DZIF Partner site Hannover, Hannover, Germany

## Abstract

*Mycobacterium tuberculosis* (*Mtb*) uses alveolar macrophages as primary host cells during infection. In response to an infection, macrophages switch from pyruvate oxidation to reduction of pyruvate into lactate. Lactate might present an additional carbon substrate for *Mtb*. Here, we demonstrate that *Mtb* can utilize L-lactate as sole carbon source for *in vitro* growth. Lactate conversion is strictly dependent on one of two potential L-lactate dehydrogenases. A knock-out mutant lacking *lldD2* (Rv1872c) was unable to utilize L-lactate. In contrast, the *lldD1* (Rv0694) knock-out strain was not affected in growth on lactate and retained full enzymatic activity. On the basis of labelling experiments using [U-^13^C_3_]-L-lactate as a tracer the efficient uptake of lactate by *Mtb* and its conversion into pyruvate could be demonstrated. Moreover, carbon flux from lactate into the TCA cycle, and through gluconeogenesis was observed. Gluconeogenesis during lactate consumption depended on the phosphoenolpyruvate carboxykinase, a key enzyme for intracellular survival, showing that lactate utilization requires essential metabolic pathways. We observed that the Δ*lldD2* mutant was impaired in replication in human macrophages, indicating a critical role for lactate oxidation during intracellular growth.

## Introduction

*Mycobacterium tuberculosis* (*Mtb*) is one of the most successful human pathogens. As reported by the WHO for 2014, 1.5 million people died from tuberculosis, while one-third of the world’s population is latently infected with *Mtb*^1^. Among other factors, the success of the pathogen relies on its ability to adapt its metabolism to the environment inside the host. A deeper understanding of the pathogen’s metabolism and its control will improve our knowledge about the pathogenicity of *Mtb*. Previous studies demonstrated that the gluconeogenic carbon flow from the tricarboxylic acid (TCA) cycle to phosphoenolpyruvate (PEP) that is mediated by the phosphoenolpyruvate carboxykinase (PckA) has a striking impact on survival of *Mtb* in macrophages and in mice^2^. Thus, during infection *Mtb* depends on carbon substrates feeding in the central carbon metabolism (CCM) down-stream of PEP. Growth on these substrates demands gluconeogenesis for biomass formation. Fatty acids, which have been demonstrated to be essential carbon sources for *Mtb*^3–5^, enter the CCM as acetyl-CoA or propionyl-CoA. Because *Mtb* is able to co-metabolize multiple carbon substrates^6^, additional gluconeogenic carbon sources might be catabolized in a PckA-dependent manner and thereby influence intracellular growth and survival.

A carbon substrate that is highly abundant at the infection site is L-lactate. Lactate is incorporated in the CCM through conversion into pyruvate, thus entering metabolism down-stream of PEP. Innate immune cells such as neutrophils, dendritic cells and macrophages produce lactate by the so-called Warburg effect^7^. After activation, those cells switch from respiration to lactate production via aerobic glycolysis, meeting the cells’ increased energy demands by rapid ATP production in glycolysis^8^. L-lactate accumulation has been detected in the lung tissue of guinea pigs infected with *Mtb*^9^. Thus, lactate is likely abundant during the early and late infection phase and might therefore present an additional carbon source for *Mtb*.

Lactate is also consumed by other intracellular pathogens during infection, as recently described for *Listeria monocytogenes*, although it is not the preferred carbon substrate^10^. The facultative intracellular pathogen *Salmonella enterica* Serovar Typhi is also able to utilize lactate as a carbon substrate^11^, indicating that lactate might indeed present a universal carbon source for intracellularly growing bacteria. For *Corynebacterium glutamicum* (*C*. *glutamicum*), a close relative of *Mtb*, L-lactate consumption is well described. This bacterium oxidizes L-lactate to pyruvate by a quinone-dependent L-lactate dehydrogenase (LldD)^12^. Interestingly, two genes encoding for potential quinone-dependent L-lactate dehydrogenase are annotated in the genome of *Mtb*, *lldD1* (Rv0694) and *lldD2* (Rv1872c)^13^.

In this study, we have analysed the lactate metabolism in *Mtb* and its relevance for growth of the pathogen. For this purpose, we have monitored lactate consumption *in vitro* and have determined pathways and fluxes during lactate metabolism. Furthermore, we characterized the lactate oxidizing potential of LldD1 and LldD2 and demonstrated that lactate consumption is important for *Mtb* to proliferate in human macrophages.

## Results

### *M*. *tuberculosis* utilizes lactate for *in vitro* growth by oxidation via the LldD2

**Figure 1:**
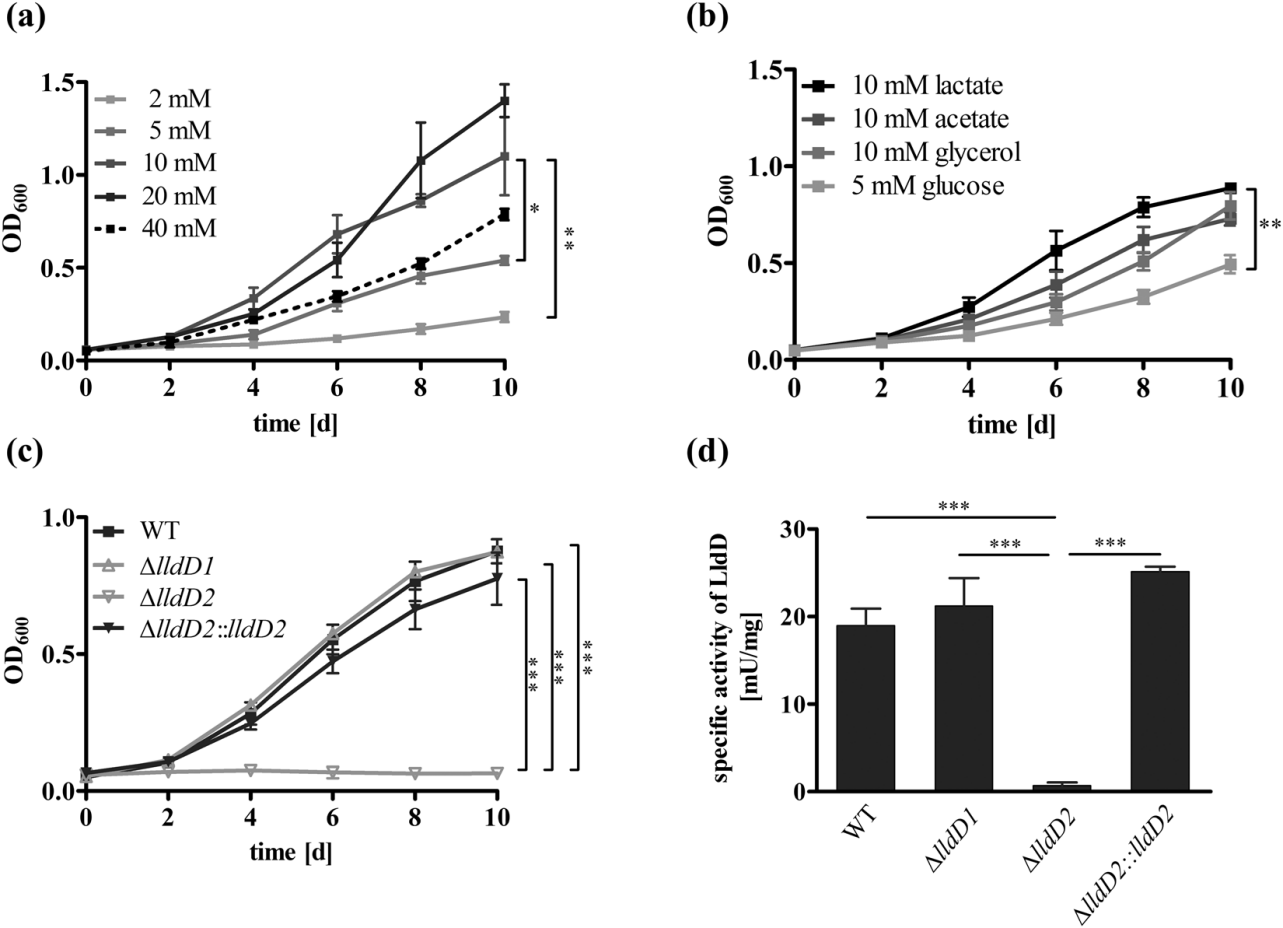
*M*. *tuberculosis* utilizes L-lactate as a carbon substrate for growth by oxidation via LldD2. (**a**) To identify the lactate concentration resulting in optimal growth of *Mtb*, lactate was offered in different amounts and the optical density was measured at the indicated time points. (**b**) To compare growth of *Mtb* on lactate and other carbon sources, *Mtb* H37Rv was grown for 10 days in the presence of lactate, acetate, glycerol, or glucose. Carbon substrates were supplied in equimolar amounts to compare their potential to induce growth of *Mtb*. (**c**) To determine the role of LldD1 and LldD2 in lactate utilization, the *Mtb* H37Rv wild type (black squares), the ∆*lldD1* and ∆*lldD2* mutant strains (grey triangles) and the complemented strain of ∆*lldD2* (black triangles) were grown on lactate as sole carbon substrate. Optical density was measured at the indicated time points. (**d**) To determine the functionality of the LldD1 and LldD2 enzymes, the *Mtb* H37Rv wild type, the knock-out mutants, and the complemented strain Δ*lldD2*::*lldD2* were grown in rich culture medium, and enzyme activity assays were performed with cell extracts using lactate as substrate. LldD activity was detected in the wild type, the Δ*lldD1* mutant and in the Δ*lldD2*::*lldD2*, while LldD activity was abolished in the Δ*lldD2* mutant. Data represent the mean of three independent experiments; error bars indicate the SEM.

To determine its impact on the metabolism of *Mtb*, L-lactate was offered as sole carbon substrate for growth (Fig. 1a). Lactate was utilized by *Mtb* for biomass formation and resulted in robust growth of the bacteria. Optimal growth was achieved in the presence of 10 mM to 20 mM lactate, and higher substrate levels did not cause a further increase in growth. In contrast, in the presence of 40 mM lactate the growth of *Mtb* was reduced, indicating that high levels of lactate have a growth inhibiting effect on the bacteria. So far, lactate levels in granulomas have not been quantified. While serum levels in healthy humans vary between 0.5 and 2 mM^14^, the lactate concentration in the lung tissue of rabbits can amount to 15 mM upon a *Staphylococcus aureus* infection^15^, and in the infected human lung lactate levels can reach up to 20 mM depending on the severity of a bacterial infection^16^.

When compared to glucose, glycerol and acetate, lactate proved to be an efficient carbon substrate for growth of *Mtb* (Fig. 1b). The bacteria showed similar growth rates in the presence of lactate as during growth on acetate or glycerol. These findings confirm the important role of gluconeogenic carbon substrates, such as lactate and acetate, for growth of *Mtb*, and demonstrate that lactate provides a potent carbon source for its metabolism.

Using a fermentative lactate dehydrogenase (Ldh), most bacteria are able to reversibly produce lactate from pyruvate and thereby regenerate NAD^+^ as a reducing equivalent^17^. However, there is no fermentative lactate dehydrogenase annotated in the genome of *Mtb*^13^. In contrast to the NADH dependent Ldh, a quinone-dependent lactate dehydrogenase (LldD) catalyses the irreversible oxidation of L-lactate to pyruvate. *C*. *glutamicum*, which is closely related to mycobacteria, has been shown to possess such an oxidative lactate dehydrogenase^18^. In the genome of *Mtb*, two genes, encoding potential quinone-dependent lactate dehydrogenases, are annotated, *lldD1* (Rv0694) and *lldD2* (Rv1872c). To analyse their impact on lactate utilization, we generated knock-out mutants, lacking either *lldD1* or *lldD2*. In the presence of lactate as sole carbon source, growth of the *lldD2* knock-out mutant was completely abolished, while the *lldD1* knock-out strain was not affected (Fig. 1c). These data indicate that LldD2 but not LldD1 is required for lactate consumption. When analysed for lactate oxidizing activity, the *lldD1* knock-out showed enzymatic activity similar to wild type levels (Fig. 1d). The total lack of enzymatic activity in the ∆*lldD2* mutant demonstrates that lactate oxidation is solely mediated by LldD2.

**Figure 2:**
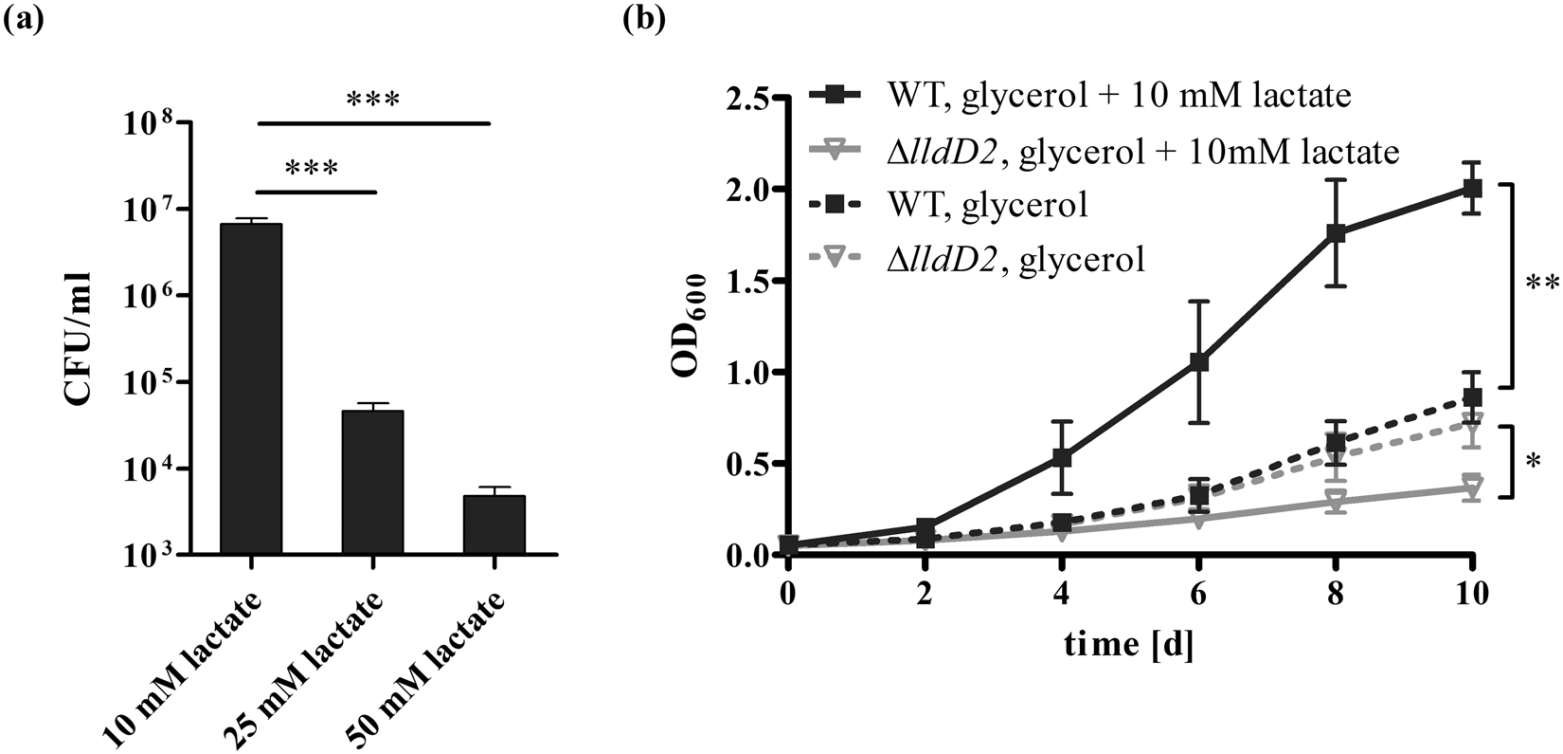
Toxicity of L-lactate on *M*. *tuberculosis*. (**a**) To analyse the effect of increasing amounts of lactate on *Mtb*, survival of the *Mtb* H37Rv wild type strain was determined on 7H10 agar plates supplemented with 10 mM, 25 mM, or 50 mM of lactate. Data represent the mean of three independent experiments; error bars indicate the SEM. (**b**) To determine whether the ∆*lldD2* mutant shows an increased sensitivity towards 10 mM lactate, *Mtb* H37Rv wild type (black lines) and the ∆*lldD2* mutant (grey lines) were grown in liquid culture in the presence of either 10 mM glycerol (dashed lines) or on a mixture of 10 mM lactate and 10 mM glycerol (solid lines). Data represent the mean of three independent experiments; error bars indicate the SEM.

We next tested if higher concentrations of lactate can be toxic for *Mtb*. We plated *Mtb* wild type on solid media (7H10 agar plates) with OADC and three different concentrations of lactate (10, 25 and 50 mM) and showed that increasing concentrations of lactate were toxic for *Mtb* (Fig. 2a). To further determine whether the inability to remove lactate causes increased stress acting on the ∆*lldD2* mutant, we analyzed the growth of the knock-out strain on a mixture of lactate and glycerol. *Mtb* wild type and the mutant were cultured in liquid media containing either glycerol, or glycerol and lactate at a non-toxic concentration (10 mM), and OD_600_ were measured at different time points. Whereas additional lactate was beneficial for the wild type and improved growth, the mutant showed less growth in the presence of lactate than in medium without lactate, suggesting that lactate becomes toxic to *Mtb* even at lower concentrations, when the organism is unable to utilize lactate (Fig. 2b).

### LldD1 is not involved in lactate oxidation in *M*. *tuberculosis*

**Figure 3:**
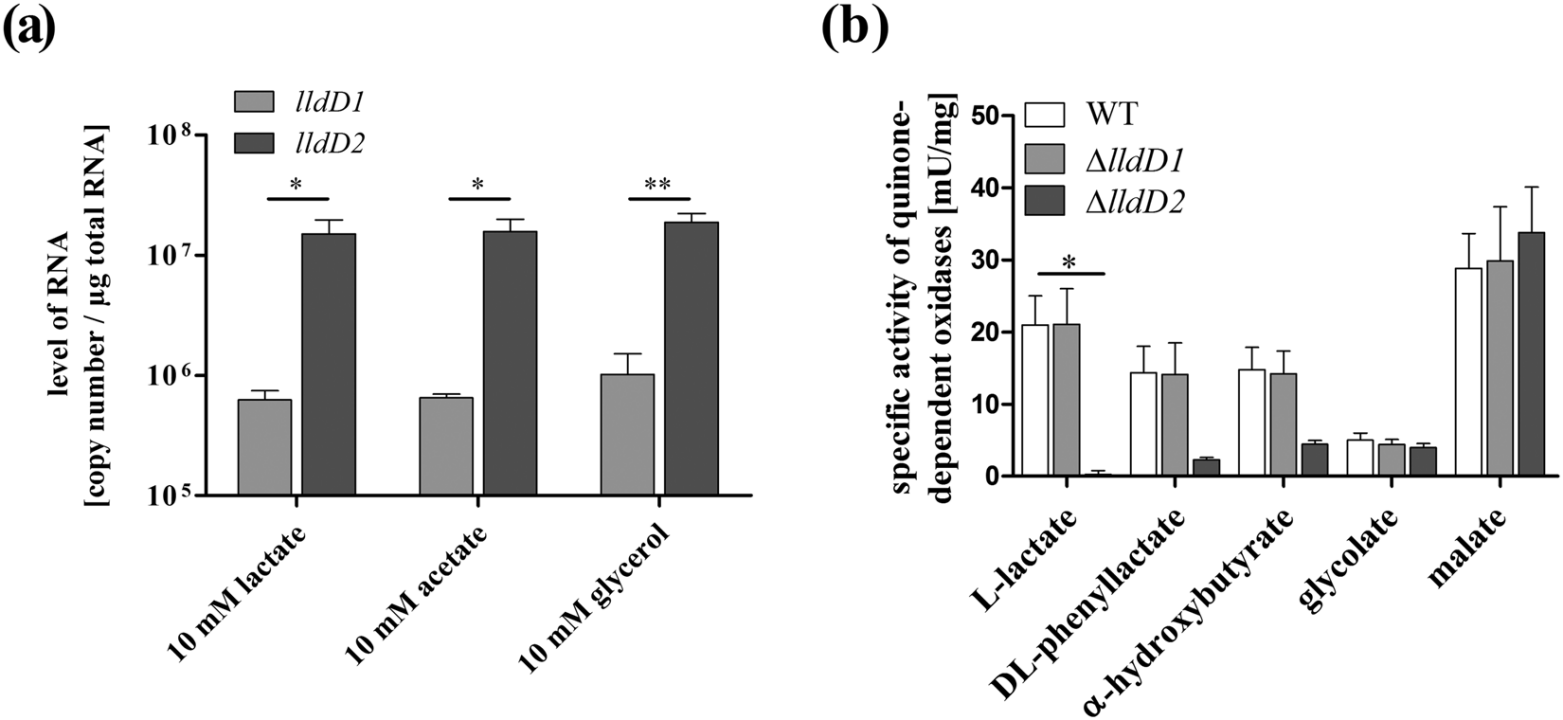
*Mtb* expresses *lldD1*, but LldD1 does not oxidize various α-hydroxy acids. (**a**) To determine whether *lldD1* is expressed in *Mtb* H37Rv growing at aerobic conditions on lactate, mRNA levels were measured by absolute quantification in RT-PCR. (**b**) To confirm the specific activity of LldD1 towards a selection of α-hydroxy acids, the *Mtb* H37Rv wild type, the Δ*lldD1* mutant and the Δ*lldD2* mutant were grown in rich culture medium, and enzyme activity assays were performed with cell extracts using lactate, DL-phenyllactate, α-hydroxybutyrate, glycolate, or malate as substrate. Enzymatic activity was detected in the wild type and the Δ*lldD1* mutant. Enzymatic activity was abolished in the Δ*lldD2* mutant on lactate, but also on DL-phenyllactate and α-hydroxybutyrate. Data represent the mean of three independent experiments; error bars indicate the SEM.

In some bacteria expression of the oxidative lactate dehydrogenase encoding gene is under the control of a lactate inducible transcriptional regulator. Therefore we examined gene expression of *lldD1* by absolute quantitative real-time PCR during growth on different carbon sources to determine if the lack of LldD1 activity resulted from repressed gene transcription (Fig. 3a). We detected approximately 8 × 10^5^ *lldD1* copies per µg of total RNA, demonstrating that *lldD1* is transcribed in the presence of lactate. Both *lldD1* and *lldD2* were expressed independently of the carbon substrate added to the medium, indicating that gene transcription is not induced by lactate. In contrast to *C*. *glutamicum*, in which the lactate dehydrogenase gene is organized in a lactate inducible operon^18^, the *lldD1* and *lldD2* genes of *Mtb* appear to be mono-cistronic, constitutively expressed genes.

LldD1 and LldD2 share only 47.4% similarity and 32.1% sequence identity and it is possible that *lldD1* encodes another quinone-dependent oxidase and thus might not be involved in lactate assimilation. Nonetheless, the highly conserved active site strongly implies that LldD1 is involved in the oxidation of small α-hydroxy acids such as lactate^19,20^. We therefore determined if LldD1 is required for the oxidation of other α-hydroxy acids. However, LldD1 was not required for oxidation of any of the tested substrates, as the ∆*lldD1* mutant showed a similar enzymatic activity as the wild type (Fig. 3b). LldD2, however, showed extended substrate specificity, being also important for oxidation of phenyllactate and α-hydroxybutyrate. A similar substrate spectrum has been reported for the oxidative lactate dehydrogenase of *Pseudomonas stutzeri*, which is involved in lactate and α-hydroxybutyrate oxidation^21^. The capacity to utilize multiple substrates might therefore be a more general feature of oxidative lactate dehydrogenases and might imply secondary functions of LldD2 in amino acid metabolism of phenylalanine, threonine and L-homoserine. Yet, the function of LldD1 remains undefined, though we were able to establish that LldD1 is not required for lactate oxidation in *Mtb*.

### Carbon flux of ^13^C_3_-lactate reveals dependency on the gluconeogenic PckA reaction

**Figure 4:**
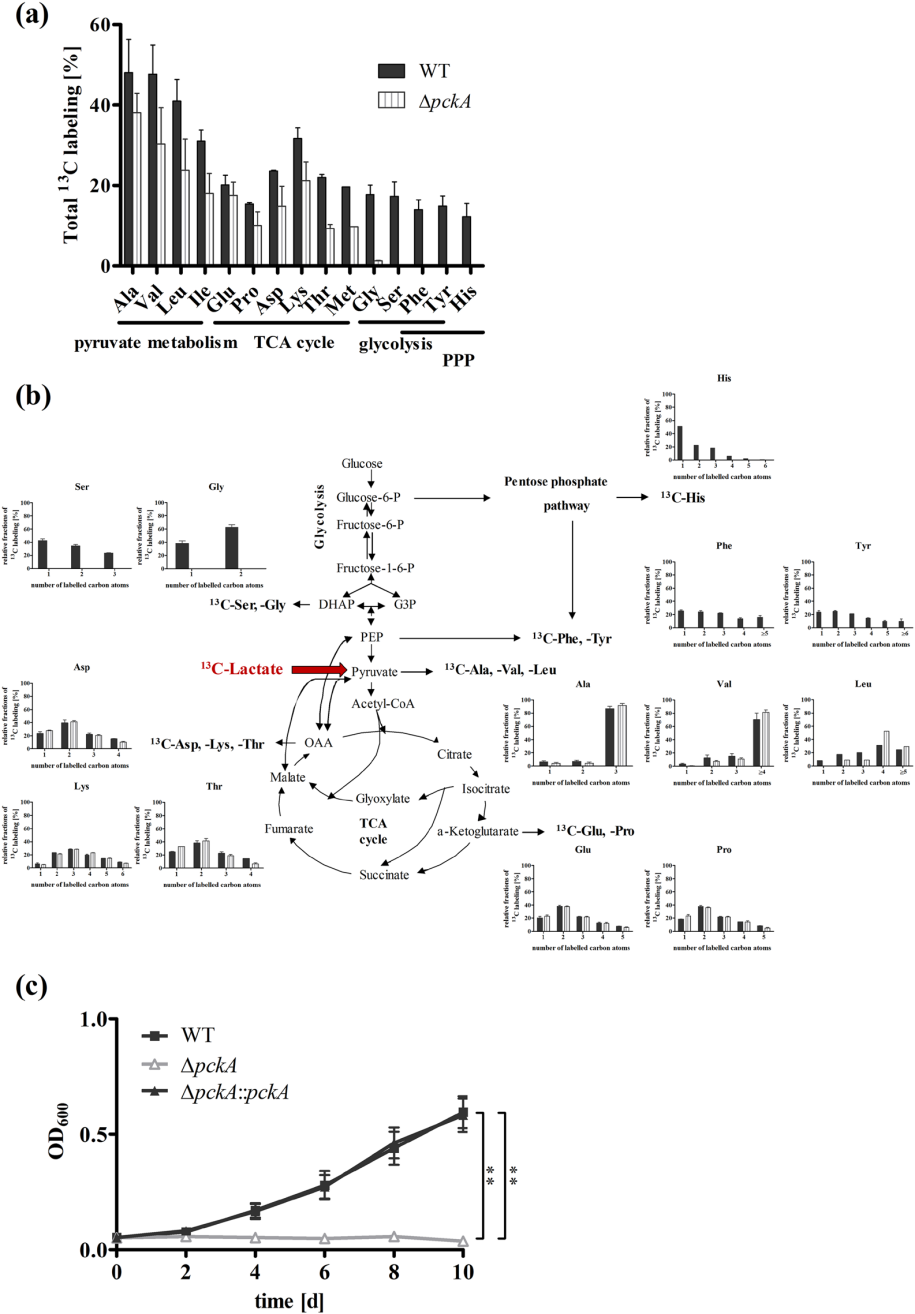
Comparison of lactate derived ^13^C-carbon flux in *Mtb* wild type and the ∆*pckA* mutant with a defect in gluconeogenesis, and growth of the ∆*pckA* mutant on lactate. To characterize the lactate derived carbon flux in *Mtb*, the *Mtb* Erdman wild type strain (black bars) and the ∆*pckA* mutant (striped bars) were grown for two days in medium containing 10 mM glucose and 10 mM [U-^13^C_3_]-L-lactate. Labelling patterns in amino acids were detected by GC/MS analysis. The overall ^13^C-enrichments are depicted in (**a**). The relative fractions (%, y-axis) of ^13^C-labelled isotopologues of the wild type strain (black bars) and the ∆*pckA* mutant (striped bars) with a given number of ^13^C-atoms (x-axis) are shown in (**b**). Data represent the mean of two independent experiments each measured in technical triplicates; error bars indicate the SEM. (**c**) To determine the impact of phosphoenolpyruvate decarboxykinase (PckA) activity on lactate utilization, growth of *Mtb* Erdman wild-type (black squares), the ∆*pckA* mutant (grey triangles) and the complemented strain (black triangles) was measured for 10 days in the presence of L-lactate as sole carbon substrate. The influence of impaired gluconeogenesis on growth was measured via optical density at the indicated time points. Data represent the mean of three independent experiments; error bars indicate the SEM.

To analyse carbon flux from lactate through the CCM, we performed isotopologue profiling. Specifically, 10 mM [U-^13^C_3_]-L-lactate was added to *Mtb* cultures growing in medium also containing 10 mM unlabelled glucose and 0.5% bovine serum albumin. After two days of growth, ^13^C-incorporation was monitored by GC-MS analysis of protein-derived amino acids following established protocols^22,23^. Label from exogenous lactate was very efficiently transferred into Ala (^13^C-excess, 48.1%), Val (47.7%) and Leu (41.0%), amino acids that are directly derived from pyruvate (Fig. 4a). Furthermore, high fractions of completely ^13^C-labelled isotopologues in these amino acids provided evidence that ^13^C_3_-lactate was efficiently taken up and directly converted into Ala, Val and Leu via ^13^C_3_-pyruvate (Fig. 4b). Amino acids synthesized from TCA cycle intermediates (Glu, Pro, Asp, Thr, Met) also acquired significant label of approximately 20% (Fig. 4a). The high fractions of two-fold labelled isotopologues in these amino acids indicated the incorporation of ^13^C_2_-labelled acetyl-CoA, which was derived from decarboxylation of three-fold labelled pyruvate (Fig. 4b). Isotopologues with three or a single labelled carbon can be explained by the activity of anaplerotic, carbon dioxide fixing reactions in lactate metabolism. Recently, fixation of carbon dioxide in *Mtb* has been described in anaplerosis, involving the activity of malic enzyme, pyruvate carboxylase and the phosphoenolpyruvate carboxykinase (PckA)^24^. PckA reversibly catalyses the production of oxaloacetate from PEP and is a known key enzyme in gluconeogenesis^2^.

Beside anaplerotic reactions, carbon from lactate was also metabolized via gluconeogenesis, as we found labelled amino acids that are synthesized from intermediates of the glycolysis (Ser, Gly, Tyr and Phe), or the pentose phosphate pathway (His, Tyr and Phe) enriched by 15–20%. Thus, we sought to further determine the role of PckA in lactate metabolism. We analysed the labelling pattern of amino acids in a *pckA* knock-out strain growing on ^13^C_3_-lactate. The ∆*pckA* mutant showed a similar labelling profile as wild type of amino acids generated from TCA cycle intermediates, indicating that the PckA reaction was not crucial for anaplerosis during lactate metabolism. However, our data also show that PckA had a strong impact on carbon flow via gluconeogenesis. While in wild type *Mtb* carbon flow from the TCA cycle was directed to gluconeogenesis, producing amino acids deriving from *de novo* generated glycolytic CCM-intermediates (^13^C-excess: Ser 17.3%, Gly 17.8%) and intermediates of the pentose phosphate pathway (^13^C-excess: His 12.3%, Tyr 14.9%, Phe 14.0%), in the *pckA* knock-out strain no labelling of gluconeogenic amino acids was detected (Fig. 4a). The complete loss of gluconeogenic carbon flow resulted in abolished growth of the ∆*pckA* mutant on lactate as sole carbon substrate (Fig. 4c). These findings demonstrate that PckA activity is essential for utilization of lactate as a carbon substrate.

### Carbon flux of ^13^C_2_-acetate reveals similarities in lactate and acetate metabolism

**Figure 5:**
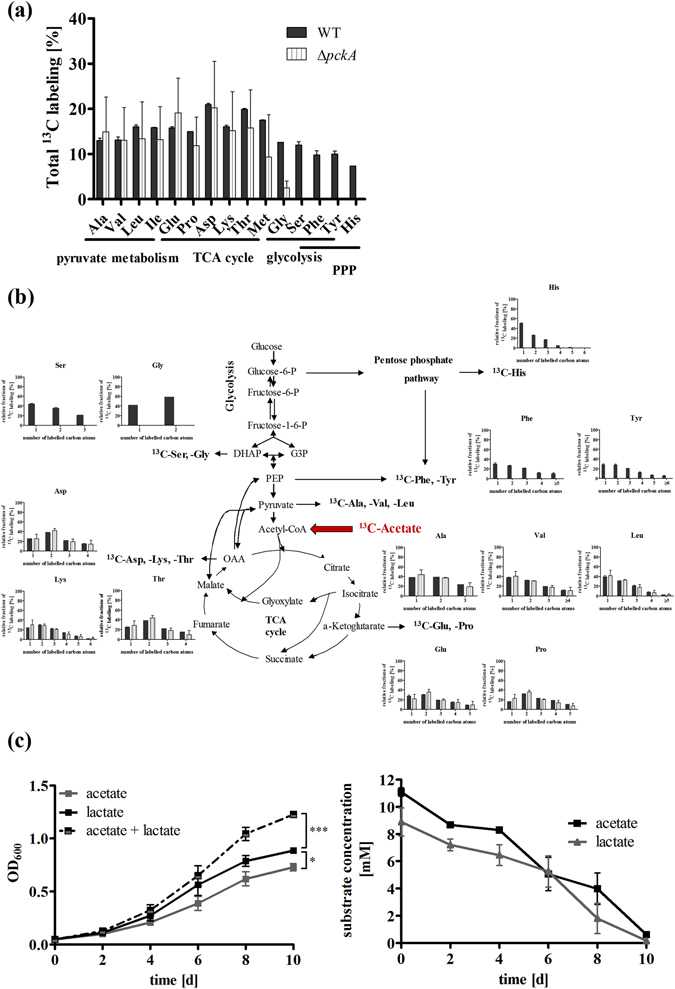
Comparison of acetate derived ^13^C-carbon flux in *Mtb* wild type and the ∆*pckA* mutant, and co-metabolism in *Mtb* wild type during growth on lactate and acetate. To characterize the acetate derived carbon flux in *Mtb*, the wild type strain (black bars) and the ∆*pckA* mutant (striped bars) were grown for two days on medium containing 10 mM glucose and 10 mM [U-^13^C_2_]-acetate. The overall ^13^C-enrichments are depicted in (**a**). The relative fractions (%, y-axis) of ^13^C-labelled isotopologues in the wild type strain (black bars) and in the ∆*pckA* mutant (striped bars) with a given number of ^13^C-atoms (x-axis) are shown in (**b**). Data represent the mean of two independent experiments each measured in technical triplicates; error bars indicate the SEM. (**c**) To analyse co-metabolism of lactate and acetate, *Mtb* H37Rv was grown in defined minimal medium containing 10 mM lactate and 10 mM acetate. Growth and substrate consumption from the medium were detected for ten days at the indicated time points. Data represent the mean of three independent experiments; error bars indicate the SEM.

Fatty acids and cholesterol are major carbon and energy sources for *Mtb in vivo*. The important role of fatty acid metabolism has been suggested to cause the growth defect of the ∆*pckA* mutant in macrophages and mice^2^. Fatty acids are degraded in β-oxidation to acetyl-CoA and propionyl-CoA entering the CCM down-stream of PEP. To determine carbon flow during growth on fatty acids, [U-^13^C_2_]-acetate, the precursor of acetyl-CoA, was provided as carbon source. Growth of *Mtb* wild type in medium containing 10 mM ^13^C_2_-acetate and 10 mM unlabelled glucose resulted in incorporation of labelled carbons in all detected amino acids (Fig. 5a). The amount of *de novo* synthesized amino acids in ^13^C_2_-acetate was reduced compared to synthesis in ^13^C_3_-lactate, implying that lactate is more efficiently used for biosynthesis. In agreement with existing knowledge^2^, we found that during growth in acetate, carbon flux is directed from acetate to the TCA cycle, requires the PckA for gluconeogenesis to produce PEP and depends on other decarboxylating enzymes, such as malic enzyme or pyruvate carboxylase, to produce pyruvate (Fig. 5b).

As lactate enters the CCM close to acetate, we expected the labelling patterns resulting from both substrates to be very similar. The patterns in amino acids synthesized from TCA cycle, glycolysis or pentose phosphate pathway intermediates were indistinguishable (Fig. 4b). Differences in carbon incorporation between lactate and acetate were only observed in the labelling profile of amino acids that use pyruvate as a direct precursor, such as alanine, valine and leucine. While the metabolism of ^13^C_3_-lactate resulted in high amounts of *de novo* synthesized alanine and valine that were fully labelled, the incorporation of the ^13^C_2_-substrate acetate into the CCM resulted in *de novo* synthesized alanine, valine and leucine that showed high amounts of one- (Ala 37.9%, Val 37.6%, Leu 38.8%) and twofold (Ala 38.7%, Val 31.9%, Leu 31.0%) labelled amino acids.

The similar growth phenotypes and the labelling patterns of amino acids synthesized from TCA cycle, glycolytic and pentose phosphate pathway intermediates in presence of ^13^C_2_-acetate or ^13^C_3_-lactate, demonstrate that upon growth on these substrates similar metabolic pathways are active. Thus, we were wondering whether both carbon substrates can be co-utilized in *Mtb*. Therefore, we supplemented the cultures with lactate and acetate and detected substrate consumption in the medium (Fig. 5c). We found that both carbon substrates were simultaneously consumed from the medium.

### Lactate oxidation is required for intracellular growth of *Mtb* in macrophages

**Figure 6:**
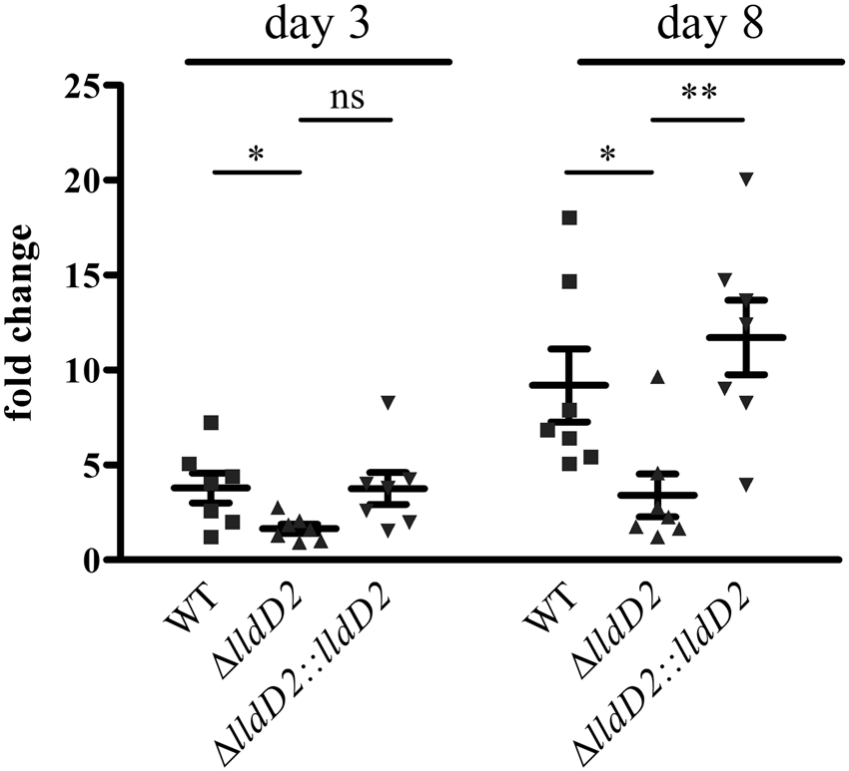
Lactate oxidation is required for optimal growth in macrophages. Human PBMC derived macrophages were infected with *Mtb* H37Rv wild type, the ∆*lldD2* mutant and the complemented strain of ∆*lldD2* at MOI 3. Macrophages were lysed four hours, three days and eight days after infection and the bacterial counts were determined. To analyze the fold change of replication, bacterial counts after three or eight days were put in relation to bacteria taken up after four hours. Data represent the mean of seven independent experiments; error bars indicate the SEM.

To further analyse the role of lactate metabolism in intracellular growth, we examined the proliferation of the wild type strain and the ∆*lldD2* mutant in human macrophages (Fig. 6). While the wild type and the complemented *lldD2* knock-out strain replicated constantly over eight days, proliferation of the *lldD2* knock-out mutant was impaired. The growth defect of the mutant demonstrates that lactate is accessible to intracellular *Mtb* and might present an additional carbon substrate for *Mtb* growing in macrophages. However, a toxic effect of accumulating lactate acting on the ∆*lldD2* mutants cannot be ruled out. Thus, L-lactate oxidation might provide a dual function in *Mtb*, by detoxifying macrophage derived lactate and by providing an additional carbon source for growth.

## Discussion

Previous studies have demonstrated that carbon metabolism of intracellular *Mtb* mainly relies on consumption of lipid components^3,4,25,26^. Furthermore, essentiality of gluconeogenesis for intracellular survival was established in *Mtb* lacking PckA activity^2^, highlighting the important role of carbon substrates oxidized in the TCA cycle and subsequently carboxylated for gluconeogenesis. To ensure robustness during infection, most pathogens choose from a variety of different carbon sources^27^. Therefore, we analysed the role of lactate, another metabolite also feeding in the TCA cycle, as an additional carbon source. In this study, we demonstrated that lactate is utilized as carbon substrate for growth of *Mtb*, and is also required for intracellular replication in human macrophages.

To metabolize lactate, *Mtb* has to gain access to this carbon substrate from the host. By stimulation of several host genes, including *ldhA*, *Mtb* increases the aerobic glycolysis in macrophages^28^. The *ldhA* gene encodes the lactate dehydrogenase A (LDHA), an enzyme mediating lactate production in most human cells. It is primarily localized in the cytosol^29^. Utilization of lactate by *Mtb* would require transport of the carbon substrate across the phagosomal membrane, as it is generally believed that the pathogen resides inside the phagosomal compartments of macrophages^30,31^, though it has been also detected in the cytosol of the host^32^. The monocarboxylate transporter 4 (MCT4), involved in lactate transport, has been recently identified on phagosomal membranes^33,34^. Furthermore, LDHA was found to be associated to the phagosomal compartments^33,34^. Thus, regardless of whether *Mtb* remains inside the phagosome or escapes into the cytosol, the combined action of the lactate dehydrogenase and the transporter might indeed expose intracellular *Mtb* to lactate.

As lactate is present at the infection site and might be accessible for intracellular *Mtb*, we analysed whether it can be utilized for carbon metabolism of *Mtb*. We have demonstrated that lactate can serve as a carbon substrate for *in vitro* growth of *Mtb*. The optimal substrate concentration for growth proved to be around 10 mM, a substrate level which can be reached in infected lung tissue^16^. We further identified the enzyme responsible in lactate oxidation. Although two quinone-dependent lactate dehydrogenases are annotated in the genome of *Mtb*^13^, we have shown that only one enzyme mediates growth on lactate and possesses lactate oxidizing activity in *Mtb*. The active lactate dehydrogenase LldD2 belongs to the family of flavin-containing enzymes, and catalyses the oxidation of lactate to pyruvate in an NAD-independent manner. The second flavin-dependent lactate dehydrogenase annotated on the genome of *Mtb* is LldD1. Even so we verified that *lldD1* was transcribed in the presence of lactate, deleting the gene had no impact on lactate oxidation in *Mtb*. For the fructose bisphosphatases (GlpX and Gpm2) of *Mtb* it has been shown that substrate specificities of paralogous enzymes can be overlapping^35^. However, we did not observe loss of enzymatic activity for various tested α-hydroxy acids in the ∆*lldD1* knock-out strain. Thus, we concluded that *lldD1* might encode for an enzyme of unknown function or might present a non-functional lactate dehydrogenase.

With respect to a functional role of the LldD2 in *Mtb*, we have shown that this enzyme is required for proliferation in human macrophages. Likewise, in murine macrophages, previous studies using a transposon site hybridization (TraSH) assay, demonstrated that a transposon mutant in *lldD2* was also impaired, but not completely attenuated^36^. In mice, however, TraSH data on survival of transposon mutants revealed that an *lldD2* mutant was only slightly affected in the first two weeks of infection^37^. On that account, the lactate dehydrogenases were so far not considered to be essential genes for survival within the host, in contrast to certain genes involved in lipid catabolism, lysine and leucine biosynthesis and gluconeogenesis^38^. While nutrient availability is limited and rather stable in the macrophage cell culture, the situation *in vivo* is more complex. The carbon availability might explain why *Mtb* lacking lactate dehydrogenase activity is impaired in macrophages but not affected in mice. The supply and amount of carbon substrates might be fluctuating with the physiological state of the host. Furthermore, granulomas contain intracellular *Mtb* in macrophages^39^ and extracellular bacteria in the liquefied or open lesions. Thus, the metabolic state of the pathogen is changing as well^40^. The metabolic composition of guinea pigs granulomas also revealed the accumulation of multiple carbon substrates, such as glycolytic carbon metabolites, amino acids, certain organic acids and lipids^9^. As *Mtb* is able to utilize these substrates for *in vitro* growth^41^, co-metabolism of multiple carbon sources, e.g. fatty acid consumption, might compensate for lactate utilization in mice.

Nonetheless, the attenuated intracellular proliferation of the *lldD2* knock-out indicates an important role for lactate oxidation in macrophages. Furthermore, similarly strong attenuation in macrophages has been described so far only for a few other metabolic mutants. Those include mutants lacking the isocitrate lyases *icl1* and *icl2*^42^ or the *pckA*^2^, demonstrating that fatty acids are oxidized by intracellular *Mtb* and highlighting the importance of gluconeogenic carbon flow from the TCA cycle. Thus, growth of *Mtb* in macrophages mainly depends on carbon substrates feeding in the CCM down-stream of PEP. These substrates include acetyl-CoA, a product of fatty acid degradation, and lactate, as we demonstrated that lactate metabolism is also dependent on PckA activity. The utilization of multiple carbon substrates feeding in the CCM down-stream of PEP might be beneficial for *Mtb* by ensuring a more robust metabolism. Indeed, we have shown that *Mtb* co-utilizes lactate and the fatty acid surrogate acetate. Thus, lactate metabolism might be able to at least partially compensate for impaired fatty acid assimilation in *Mtb* knock-out mutants. The impaired survival of a mutant lacking the *icl* genes might rather rely on the formation of toxic intermediates of the methylcitrate cycle than on carbon restriction^43,44^.

A mixed diet including fatty acids and C_3_ substrates, entering the CCM at the site of pyruvate and glycerol-3-phosphate, was recently suggested for metabolism of *Mtb* growing in THP-1 macrophages^45^. Alanine was demonstrated to be a likely C_3_ metabolite received from the macrophages. By deamination and oxidation alanine can be converted into pyruvate by an alanine dehydrogenase^46^. We have shown that pyruvate is also produced by lactate oxidation and therefore the same metabolic pathways should be active upon lactate and alanine consumption. Thus, it seems possible that next to fatty acids multiple carbon substrates feed in the CCM down-stream of PEP and are co-utilized by *Mtb*.

The co-utilization of multiple carbon substrates would result in a robust intracellular metabolism that would not be impaired, when lactate is restricted. Still, replication of *Mtb* lacking the lactate oxidizing enzyme in macrophages was attenuated. Growth inhibition might rather result from toxic effects of accumulating lactate as lactate is known to increase oxidative stress in the Fenton reaction^47^. Furthermore, the flavin-dependent lactate dehydrogenases are widely distributed among catalase-positive bacteria, to reduce lactate levels in the presence of ROS^17^. As infected macrophages produce increased amounts of ROS and lactate, lactate oxidation to pyruvate by *Mtb* might play a role in defence against oxidative stress. Lactate oxidation by LldD2 might therefore possess a dual function, to remove mounting concentrations of lactate and to provide additional carbon substrates for metabolism.

In conclusion, we report that lactate provides a carbon and energy source for *Mtb*. It is oxidized to pyruvate by the quinone-dependent LldD2. As has been recently shown for fatty acids, lactate metabolism depends on phosphoenolpyruvate carboxykinase for gluconeogenesis. Furthermore, the ^13^C-profiles of amino acids during growth on lactate and the fatty acid surrogate acetate revealed strong similarities in lactate and fatty acid metabolism. Both, lactate and acetate were shown to be co-utilized by *Mtb*, probably ensuring a broader and more robust carbon metabolism of *Mtb*. Indeed, lactate oxidation proved to be required for proliferation in human macrophages. Thus, lactate provides an efficient carbon substrate for growth and its oxidation is beneficial for intracellular proliferation.

## Methods

### Strains and media

To gain substantial growth of *Mtb* H37Rv (ATCC 25618) and its derivative mutants, the strains were cultured in Middlebrook 7H9 liquid medium (Difco Laboratories, Detroit, MI, USA) supplemented with 0.5% bovine serum albumin fraction V, 14 mM sodium chloride, 10 mM D-glucose, 50 mM glycerol, and 0.05% tyloxapol. Middlebrook 7H10 agar supplemented with 5.8 mM sodium chloride, 0.2% bovine serum albumin fraction V, 4.4 mM glucose, 50 mM glycerol, 0.0001% catalase and 0.06% oleic acid was used as solid medium. To detect growth on different carbon substrates and to determine substrate consumption, experiments were performed in a chemically defined liquid medium^25^ containing 7.3 mM KH_2_PO_4_, 17.6 mM Na_2_HPO_4_, 190.8 µM ferric ammonium citrate, 2 mM MgSO_4_, 3.4 µM CaCl_2_, 0.35 µM ZnSO_4_, 50 mM asparagine, 0.05% tyloxapol and the respective carbon source in the indicated concentrations. In cultures containing the complemented strain, 50 µg/ml hygromycin B was supplied. All experiments were performed at well-aerated, fast growing conditions at 70 rpm and 37 °C.

### Generation and complementation of ∆*lldD1* and ∆*lldD2* deletion mutants in *Mtb*

The strains Δ*lldD1* (MAS38) and Δ*lldD2* (ND12) were generated via homologous recombination^48^ in *Mtb* H37Rv. Therefore, cosmids containing the *lldD1* (Rv0698) or the *lldD2* (Rv1872c) gene were isolated via colony blot hybridization from an *Mtb* H37Rv cosmid library^49^. The respective genes were subcloned in vector pBSK(−). An unmarked 538 bp in-frame deletion was generated in *lldD1* and 918 bp were in-frame deleted in *lldD2*. Deleted *lldD1* and *lldD2* with flanking regions of approximately 1,000 bp were inserted into a suicide plasmid pYUB657^48^ containing a hygromycin resistance cassette and the *sacB* gene, encoding a levansucrase, for negative selection. By counter-selection on 7H10 agar-plates containing 2% sucrose clones were obtained that had undergone double cross-over. The resulting mutant strains were validated by Southern blot hybridization. Polar effects of the deletions on genes downstream of *lldD1* or *lldD2* are unlikely as all deletions were set in frame. Δ*lldD2* was complemented with an integrating vector, pMV306.hyg^50^, carrying the *lldD2* gene and an additional 200 bp genomic region upstream of the gene containing the putative promoter region. The complemented mutant strain is termed Δ*lldD2*::*lldD2* (SB17). The *ΔpckA* knock-out strain in *Mtb* Erdman^51^ and the parent Erdman wild type strain were kindly provided by Sabine Ehrt.

### Determination of quinone-dependent enzyme activity

For determination of enzyme activities, exponentially growing cells were harvested by centrifugation (3,000 g, 10 min, 4 °C) and washed twice with 50 mM ice-cold phosphate buffer, pH 7.0. Cell extracts were prepared by resuspension in 1 ml of 50 mM phosphate buffer, pH 7.0, and homogenization in a Mini-Beadbeater-8 (BioSpec Products, Bartlesville, USA). After centrifugation at 4 °C for 10 min at 16,200 g, enzyme activity was immediately determined in the cell debris-free supernatant. L-Lactate dehydrogenase activity was detected by a modified assay according to Molinari and Lara^52^. Assays (volume, 1 ml) contained 100 mM phosphate buffer (pH 7.5), 50 µM 2,6-dichloroindophenol (DCPIP) and 20 mM L-lactate. To determine the substrate specificity of the lactate dehydrogenases, malate, glycolate, DL-phenyllactate, or α-hydroxybutyrate were offered to the cell extracts in final concentrations of 20 mM. The reaction was started by addition of 20 µl of cell debris-free supernatant as described above. Enzyme activity was assayed spectrophotometrically by determining the decrease in absorbance of DCPIP (ε_600nm_ = 20 cm^2^ µmol^−1^).

### Absolute quantification by real-time PCR

RNA was extracted from *Mtb* grown in a chemically defined medium containing either 10 mM lactate or 10 mM glycerol for 5 days. Subsequently, the cultures were incubated with an equal amount of 5 M GTC buffer (5 M guanidinium isothiocyanate, 0.5% *n*-laurylsarcosine, 0.7% sodium citrate, 0.7% β-mercaptoethanol) at room temperature for 15 min. Following centrifugation at 3,500 g for 15 min, the pellet was resuspended in 1 ml of TRIzol (Invitrogen, Germany). Cell disruption was performed in the Hybaid RiboLyser (Hybaid, Teddington, UK). Cell debris was removed by centrifugation and the supernatant was used for chloroform extraction. Following nucleic acid extraction, RNA was purified using the RNeasy Kit (Qiagen, Hilden, Germany). An optional on-column DNaseI digestion for 1 h was included to improve the removal of genomic DNA. Concentration and purity of RNA yield was spectrophotometrically determined in the NanoDrop 1000 (Thermo Scientific, USA). 2 μg of total RNA and known amounts of template RNA (10^4^–10^11^ copies) were transcribed into cDNA using the SuperScript® II reverse transcriptase (Invitrogen), followed by cleaning with the QIAquick PCR Purification Kit (Qiagen, Hilden, Germany). Control reactions without SuperScript® II reverse transcriptase in the transcription reaction (no template controls (NTCs)) were used to estimate the amount of template DNA carryover in the final RNA preparation. TaqMan® real-time PCR was used for the quantification of *lldD1*, *lldD2* mRNA, NTCs and cDNA standards, using a custom gene expression assay (Applied Biosystems, Foster City, CA, USA). A standard curve, plotted from cDNA of known amounts of template RNA (10^4^–10^11^ copies), was used to calculate the amount of RNA transcript from the *in vitro* transcription reaction.

### Labelling Experiments

*Mtb* Erdman strain and the derivative Δ*pckA* mutant were cultured in 7H9 broth supplemented with 0.5% bovine serum albumin fraction V, 14 mM sodium chloride, 0.05% tyloxapol, 10 mM D-glucose. 10 mM [U-^13^C_3_]lactate (Promega) or 10 mM [U-^13^C_2_]acetate (Promega) were added, respectively. After 5 days, 10^8^–10^9^ cells were centrifuged (3,000 g, 10 min) and the cell pellet was washed 2× with PBS and was autoclaved at 121 °C.

For isotopologue profiling and to determine the ^13^C-excess values of amino acids, bacterial cells (approximately 10^9^ cells) were hydrolysed in 0.5 ml of 6 M HCl for 24 h at 105 °C, as described before^23^. The HCl was removed under inert atmosphere, and the remainder was dissolved in 200 μl acetic acid. Purification of the hydrolysate was performed on a cation exchange column of Dowex 50Wx8 (H^+^ form, 200–400 mesh, 5 × 10 mm). The column was washed with 1 ml 70% methanol and 1 ml of ultrapure water. Elution of the column was conducted with 2 ml distilled water and 1 ml 4 M ammonium hydroxide. A 0.5 ml aliquot of the eluate was dried under a nitrogen stream at 70 °C. Subsequently, the remainder was dissolved in 50 μl water-free acetonitrile and 50 μl *N*-(tertbutyldimethyl-silyl)-*N*-methyl-trifluoroacetamide containing 1% tert-butyl-dimethylsilylchlorid (Sigma). The mixture was kept at 70 °C for 30 min. Resulting tert-butyldimethylsilyl derivates (TBDMS) of amino acids were further analysed by GC/MS on a GCMS-QP2010 Plus Gas Chromatograph/Mass Spectrometer (Shimadzu) equipped with a fused silica capillary column (Equity TM-5; 30 m × 0.25 mm, 0.25 μm film thickness; SUPELCO) and a quadrupol detector. One μl of a TBDMS derivative solution was injected in a 1:10 split mode on the column at an interface temperature of 260 °C and a helium inlet pressure of 70 kPa. This was followed by development of the column at 150 °C for 3 min with a temperature gradient of 7 °C/min to a final temperature of 280 °C that was maintained for 3 min. Data were collected using the LabSolutions software (Shimadzu). All samples were measured three times (technical replicates) and isotopologue compositions were calculated as described before^22^.

### Determination of lactate and acetate concentrations

During cultivation of *Mtb* H37Rv wild type strain in a chemical defined medium, samples were collected to determine L-lactate and acetate concentrations in the medium. After centrifugation of the sample (16,200 g, 10 min) the concentrations of L-lactate and acetate in the supernatants were determined enzymatically (r-biopharm, Germany).

### Isolation and differentiation of primary human monocytes

Peripheral blood mononuclear cells (PBMCs) were isolated from buffy coats (purchased from the Red Cross Blood Donation Center in Springe, Germany) by Ficoll gradient centrifugation. Monocytes were adhered to 24-well plates (5 × 10^5^/well) and differentiated in RPMI supplemented with 2 mM glutamine, 10% fetal calf serum (FCS), 10,000 U/ml penicillin, 10 mg/ml streptomycin and 10% human serum for 10 days at 37 °C with 5% CO_2_.

### Macrophage infection

Human macrophages were infected with *Mtb* H37Rv wild type, the Δ*lldD2* mutant and the *lldD2* complemented knock-out strain (multiplicity of infection (MOI) of ∼3). *Mtb* strains were grown in 7H9 broth to mid-log phase. The 7H9 broth was completely removed by centrifugation (3,000 g, 10 min) and bacteria were washed thrice with PBS. The pellets were resuspended in RPMI medium containing 2 mM glutamine, 10% FCS and 10% human serum. Macrophages were infected with an MOI 3 and incubated for 4 h at 37 °C with 5% CO_2_. This incubation period was followed by removal of the infection medium. Macrophages were washed three times with PBS to remove extracellular bacteria and culture medium was added to the cells. The cultures were then incubated for a subsequent 3 or 8 days. Following incubation with *Mtb*, the cells were lysed by incubation in 0.5% Triton X-100 (Sigma-Aldrich, St. Louis, MO) for 10 min at 37 °C. The lysate was serially diluted and plated on Middlebrook 7H10 to determine the number of colony forming units (CFUs).

### Statistical Analysis

One-tailed unpaired Student’s t-test was used for the analysis of differences between two groups (Figs 2 and 6), whereas for differences between three or more groups statistical significance was tested using one-way ANOVA with Tukey’s multiple comparison test (Figs 1, 4c and 5c). Statistical significance is depicted as *p < 0.05, **p < 0.01, or ***p < 0.001.
