# Author Correction: Lactate oxidation facilitates growth of *Mycobacterium tuberculosis* in human macrophages

**DOI:** 10.1038/s41598-018-23446-8

**Published:** 2018-03-22

**Authors:** Sandra Billig, Marie Schneefeld, Claudia Huber, Guntram A. Grassl, Wolfgang Eisenreich, Franz-Christoph Bange

**Affiliations:** 1 Dept. of Medical Microbiology and Hospital Epidemiology, Hannover Medical School, Hannover, 30625 Germany; 2 Dept. of Biochemistry, Technische Universität München, Garching, 85747 Germany; 3 DZIF Partner site Hannover, Hannover, Germany

Correction to: *Scientific Reports* 10.1038/s41598-017-05916-7, published online 25 July 2017

This Article contains a typographical error in the Methods section under subheading ‘Generation and complementation of ∆*lldD1* and ∆*lldD2* deletion mutants in *Mtb*’ where,

“Therefore, cosmids containing the *lldD1* (Rv0698) or the *lldD2* (Rv1872c) gene were isolated via colony blot hybridization from an *Mtb* H37Rv cosmid library^49^.”

should read:

“Therefore, cosmids containing the *lldD1* (Rv0694) or the *lldD2* (Rv1872c) gene were isolated via colony blot hybridization from an *Mtb* H37Rv cosmid library^49^.”

